# Conceptualizing monetary benchmarks for health investments toward poverty reduction in low- and lower middle-income countries

**DOI:** 10.1371/journal.pgph.0000487

**Published:** 2022-06-17

**Authors:** Averi Chakrabarti, Stéphane Verguet

**Affiliations:** Department of Global Health and Population, Harvard T.H. Chan School of Public Health, Boston, Massachusetts, United States of America; University of Ottawa, CANADA

## Abstract

Public spending can improve population well-being, for example, by averting or reducing poverty. We aim to conceptualize monetary benchmarks for health sector investments oriented towards poverty alleviation in low- and lower middle-income countries. These benchmarks are meant to indicate the approximate range of health sector costs incurred to avert a single case of poverty across countries. Such conceptualizations could help identify the health interventions that are worthwhile investing in from financial risk protection and social welfare standpoints. We sourced secondary data from the World Bank for low-income and lower-middle-income countries over 2002–2019, including: per capita government expenditures on health, the proportion of a country’s population living under the international poverty line ($1.90 per day, 2011 Purchasing Power Parity), and the features of national social protection programs whose primary intent is poverty reduction. We then examined the associations between poverty headcount and per capita government health spending to gauge the potential relationship between this spending and poverty reduction. Subsequently, we derived a range of plausible poverty reduction benchmarks (PRBs). We also computed the per capita costs of national poverty reduction programs so as to contrast these with the estimated range of PRBs. Priority setting in low- and lower-middle-income countries could be informed by health-sector PRBs, in addition to burden of disease and cost-effectiveness considerations. The computed PRBs, expressed in dollars per poverty case averted, can possibly be viewed in a manner akin to economic evaluation thresholds which are usually expressed in dollars per disability-adjusted life year averted.

## Introduction

Much progress has been made towards global poverty reduction over the past two decades. While 1.9 billion people (36% of the global population) were living in poverty (on less than $1.90 per day, 2011 Purchasing Power Parity (PPP)) in 1990, the number declined to 700 million (9%) in 2017 [1]. This kind of progress has occurred across the board, including in low- and middle-income countries, that went from having 45% of their population in poverty in 1990 to 11% in 2017 [1]. The COVID-19 pandemic has, however, interrupted this trajectory: as many as 115 million additional people were likely pushed into poverty in 2020 alone [2]. The impoverished have low levels of human capital (poor education and health), lack access to basic needs such as healthcare and sanitation, and are highly vulnerable to natural disasters, physical insecurity, and catastrophes [3]. These overlapping deprivations tend to make it difficult for impoverished individuals to escape poverty, and can essentially operate as poverty traps [4]. Crises like COVID-19 are likely to further dampen prospects for social mobility among the poor and the vulnerable, and to weaken their resilience to future negative events [2].

Moving people out of poverty is a priority for national governments and has gained greater importance in light of the COVID-19 pandemic. Ending poverty in all its forms everywhere by 2030 is the foremost United Nations (UN) Sustainable Development Goal (SDG) [5]. In advancing towards this end, it is critical to identify the policies and interventions that have significant poverty alleviation returns on investment, in other words, those that have poverty reduction benefits that are large relative to the costs incurred. Policymakers could then implement the interventions that have the potential to avert the maximum amount of poverty per dollar of budget expenditure.

The current analysis is motivated by a desire to leverage the poverty alleviation potential of national health systems. Out-of-pocket (OOP) health expenditures are a major driver of financial stress and impoverishment. In 2017, 70 million individuals or 1% of the global population were pushed below the poverty line due to OOP health expenditures [1]. This burden can be particularly high in low- and middle-income countries, with more than 3% of some country populations becoming impoverished in a year due to health spending [6]. The SDGs envision the provision of health-related financial risk protection for all through universal health coverage [5]. Additionally, targeted investments channeled through national health systems, including insurance programs and pre-payment mechanisms, can be designed to prevent impoverishment from OOP medical spending [6,7]. Public health spending can accordingly be instrumental in reducing poverty even though that is not the primary aim of the health sector. Government health spending on basic services (e.g. primary care) can also reduce health inequities by bringing all individuals within the reach of essential healthcare [8]. As the COVID-19 pandemic has shown, poor and vulnerable populations bear disproportionate risks of ill health as well as the consequent financial repercussions. Accordingly, it is important to have health sector investments be guided by their ability to guard against poverty.

In this analysis, we look at the association between country-level health spending and poverty in low- and lower-middle-income countries. Estimates of this relationship can help in understanding the potential poverty reduction benefits of national health systems that are delivered in conjunction with health services, and can thereby motivate further investments into national health systems. Some interventions may or may not present the best value for money. In other words, the cost of lifting people out of poverty for some interventions or policies could be too high. Therefore, it may be valuable to define a monetary benchmark for interventions toward poverty reduction. We refer to the cost per poverty case averted in a set of countries as a “poverty reduction benchmark” (or PRB). These PRBs could then be used as a standard to gauge whether an intervention is worthwhile investing in (or not) from financial risk protection and social welfare standpoints. For example, if the costs per poverty case averted for an intervention are much higher than a given PRB, it might be worth considering alternative interventions that potentially would engender more value for money (with lower costs per poverty case averted).

In this spirit, we can start by examining the associations between public health expenditures and poverty rates in low- and middle-income countries to roughly approximate PRBs. We restrict our inquiry to public expenditures on health, a major sector that governments consistently earmark funds for and for which data are routinely reported and available across countries. Also, public health spending can be broadly viewed as comprising efforts aimed at enhancing the level and distribution of population wellbeing. Due to these reasons, studying the association between public health spending and poverty rates might be helpful for estimating PRBs. Similarly, one could also examine the costs and potential poverty alleviation impact of social protection programs with a direct intent of reducing poverty, in order to infer the distribution of such costs per poverty case averted across countries. Comparing the latter distribution with the PRB estimates obtained for the health sector could provide a sense of whether seeking to provide financial risk protection through the health sector might be a “good buy” in terms of poverty reduction. In this paper, we discuss and draw from these possible approaches to conceptualize and compute PRBs.

## Possible analytical approaches

### The association between government health expenditures and poverty rates

We sourced data from the World Bank’s World Development Indicators (WDI) database [1]. Given our focus on poverty, we restricted our analysis to countries that have high poverty rates–countries catgorized as low-income and lower-middle-income countries under the World Bank’s country income group classification [9]. For poverty, we used the proportion of a country’s population living on less than $1.90 per day (2011 PPP; the international poverty line). For government/public health expenditures, we used the per capita domestic general government health expenditures (constant 2017 international dollars).

We relied on public health spending as these data were readily available for a large number of countries over time. As contextual indicators, we also extracted from the WDI database: country gross domestic product (GDP) per capita (in constant 2017 international dollars) and the Gini index. All data used were country-level annual estimates. Since data for the main variables of interest (e.g. poverty headcount, per capita public health spending) were consistently available since 2000, we restricted our study sample to the years 2000 onwards.

Overall, our study sample included countries that had data on all variables of interest for at least two years since 2000. We had a total of 63 countries, each of which had between 2 and 18 years with complete information (spanning the years between 2002 and 2019): 20 low-income and 43 lower middle-income countries (see Table A in S1 Text; analyses with upper middle-income countries are also reported for completion in Tables D-F in S1 Text). We present summary statistics, separately for the first and last year of available data for each country (Table 1). Across all country categories and over the time period studied, the poverty headcount decreased while per capita public health expenditures increased.

**Table 1:** Summary statistics of the sample of low- and lower-middle-income countries.

	Initial mean	Final mean
Poverty headcount (proportion of country population)	0.39	0.28
*Low-income*	0.61	0.47
*Lower-middle-income*	0.28	0.19
Government health expenditures per capita lagged one year ($)	63	100
*Low-income*	21	26
*Lower-middle-income*	82	134
GDP per capita ($)	3,290	4,597
*Low-income*	1,388	1,775
*Lower-middle-income*	4,174	5,910
Gini index	41	40
*Low-income*	40	40
*Lower-middle-income*	41	40
*N*	*63*	
*Low-income*	*20*	
*Lower-middle-income*	*43*	

Notes: The poverty line is defined by $1.90 per day in 2011 international prices. Government health expenditures per capita and gross domestic product (GDP) per capita are adjusted for purchasing power parity and are in constant 2017 international dollars.

First, we examined the relationship between per capita public expenditures on health and poverty headcount, in the following way:

$$ln({Pov}_{c,t})=\alpha_{c}+\beta *ln({PHE}_{c,t-1})+\gamma *X_{c,t}+\varepsilon_{c,t},$$ (1)

where ln(*Pov*_*c*,*t*_) is the natural logarithm of the proportion of the population in poverty in country *c* in year *t*, *α*_*c*_ are country fixed effects that capture all time-invariant country-specific characteristics (e.g. features unique to a country), ln(*PHE*_*c*,*t*−1_) is the natural logarithm of per capita government health expenditures in country *c* in year *t*−1, *γ* is a vector of country characteristics (including GDP per capita (logged) and Gini index), and *ε*_*c*,*t*_ is the error term. We use lagged expenditures (*t*−1) since the potential consequences of investments are not likely to be realized immediately. *β* is the coefficient of interest: it indicates the percent change in the proportion of the population in poverty when lagged per capita public expenditures on health go up by one percent. We estimated Eq (1) for both the entire sample of countries, and also separately for low-income and lower middle-income countries. As a sensitivity analysis, we also estimated Eq (1) using public health expenditures lagged by five years as the main covariate (i.e. *PHE*_*c*,*t*−5_) since poverty levels might respond to public spending with a greater time lag.

Next, we utilized the *β* coefficient from Eq (1) to quantify the associated change in poverty when per capita government health expenditures increases. In doing so, we used the last year of data on poverty headcount and per capita public health spending available per country. Specifically, if country A has *N* as its total population and *p* proportion of its population in poverty, a one-percent increase in spending would change the number of people living in poverty by $\frac{\beta }{100}\times p\times N$. We also estimated the budget amount corresponding to a one-percent increase in government health expenditures. By dividing the change in expenditures by the change in poverty, it is possible to derive a monetary cost of poverty reduction. This is essentially the cost of averting one case of poverty through the health sector and would represent the country-level PRB.

We accounted for estimation uncertainty (from the model in Eq (1)), through sampling *n* = 1000 *β* values extracted from a multivariate normal distribution using mean and variance-covariance matrices from the fitted model. This resulted in *n* PRB estimates for each country. To provide a sense of the distribution of estimates for countries (separately for low-income and lower middle-income countries), for each of the *n* iterations, we derived an average PRB estimate across countries. From these *n* averages, we extracted the median, and the 2.5 and 97.5 percentiles to determine 95% uncertainty ranges (URs) for PRBs.

### Costs and benefits of poverty reduction programs

We also reviewed social protection programs from low- and middle-income countries aimed at reducing poverty, drawing from the World Bank’s Atlas of Social Protection Indicators of Resilience and Equity (ASPIRE) database [10]. These indicators provide an overview of social assistance, social insurance and labor market programs at the country level by sourcing information from the administrative records of programs and national household surveys. The ASPIRE data might not capture the full universe of social protection programs within countries and data availability limitations render the indicators not wholly comparable across countries [10]. Despite such shortcomings, the ASPIRE data are valuable in the absence of alternative cross-country sources of information on social protection.

We used the following indicators from the ASPIRE database: social protection and labor (SPL) system coverage, average transfer amounts (in PPP terms) and poverty headcount reduction achieved by SPL programs. For each indicator, we used the last year available per country for a total of 33 countries (low-income and lower-middle income countries only). Combining these data with population estimates [1] allowed us to estimate the distribution of SPL program costs per poverty case averted within the entire sample of countries and separately by income category. Estimates of SPL program costs per case of poverty averted could then be compared to those obtained for the health sector (the estimated PRBs). Such a comparison can provide some intuition of whether the financial protection benefits provided via health sector investments might represent a “good buy” in terms of poverty reduction.

All analyses were conducted using R (version 3.6.2) and STATA (version 14).

### Research ethics approval

The study did not involve human participants and used only secondary anonymous data. Thus ethics approval was not obtained.

## Tentative estimates of PRBs

We begin by estimating the association between government health spending and poverty using a sparse version of specification (1)—essentially a bivariate model—and then gradually add in controls. These results are presented in Table 2. The preferred specification (including country fixed effects and controls) indicates that a 1.0% increase in government health spending would be associated with roughly a 0.5% reduction in the national poverty headcount ratio (column 3). Splitting the sample into different income groupings (Table 3) shows that the coefficient is higher in magnitude for countries in the lower-middle-income category.

**Table 2:** Estimating the association between government health spending and poverty alleviation—low-income and lower-middle-income countries.

	(1)	(2)	(3)
Dependent variable:	Log poverty headcount ratio		
			
Log government health spending (lagged one year)	-0.983**	-1.398**	-0.458**
	(0.059)	(0.130)	(0.125)
Log GDP per capita			-2.452**
			(0.239)
Gini index			0.054**
			(0.010)
Country fixed effects		X	X
Observations	273	273	273
R-squared	0.51	0.87	0.93
# countries	63	63	63

Standard errors in parentheses. Statistical significance

***p<0.01

**p<0.05

*p<0.10.

GDP = gross domestic product. Data covers the years 2002–2019. Source: World Development Indicators.

**Table 3:** Estimating the association between government health spending and poverty alleviation—differences across income groupings.

	(1)	(2)
Income classification:	Low-income	Lower-middle-income
Dependent variable:	Log poverty headcount ratio	
Log government health spending (lagged one year)	-0.296**	-0.432**
	(0.108)	(0.161)
Log GDP per capita	-0.851**	-2.893**
	(0.234)	(0.299)
Gini index	0.034**	0.052**
	(0.011)	(0.011)
Country fixed effects	X	X
Observations	60	213
R-squared	0.91	0.92
# countries	20	43

Standard errors in parentheses. Statistical significance

***p<0.01

**p<0.05

*p<0.10.

GDP = gross domestic product. Data covers the years 2002–2019. Source: World Development Indicators.

In sensitivity checks, we show that results are fairly consistent when we use government spending lagged by five years as the main covariate (Table B in S1 Text), and add external health spending to government health spending (Table C in S1 Text) (from the World Bank). Given our focus on the poorest countries, we did not include upper-middle-income countries in our sample. For completeness however, we also present estimates for upper-middle-income countries in Tables D and E in S1 Text; the main relationship of interest fails to attain statistical significance in this sub-sample (Table F in S1 Text, Column 1).

Using the estimated associations (Tables 2 and 3), we compute the costs of averting a poverty case via government health spending. As discussed above, we use the last year of health spending and poverty headcount data per country. The mean cost to avert a poverty case through health spending in all countries in the sample would be about $44,000 (95% UR: $28,000–94,000); for low-income countries, it would be $460 ($250–1,600), and for lower-middle-income countries it would be about $68,000 ($38,000–255,000).

**Fig 1:**
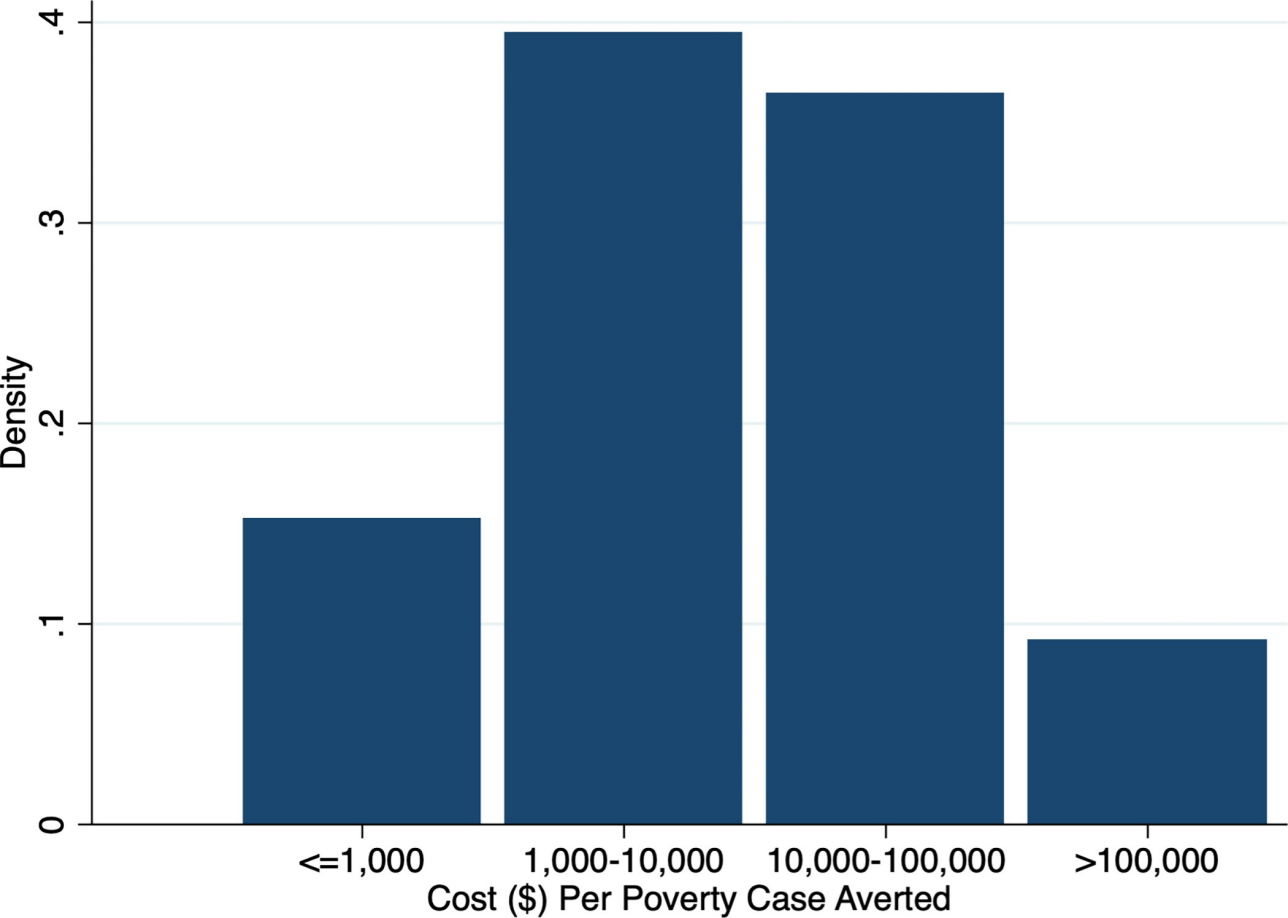
Distribution in estimated costs ($) per poverty case averted across countries using social protection program data. Notes: Source: The World Bank Atlas of Social Protection Indicators of Resilience and Equity (ASPIRE). Sample includes low-income and lower-middle-income countries.

**Fig 2:**
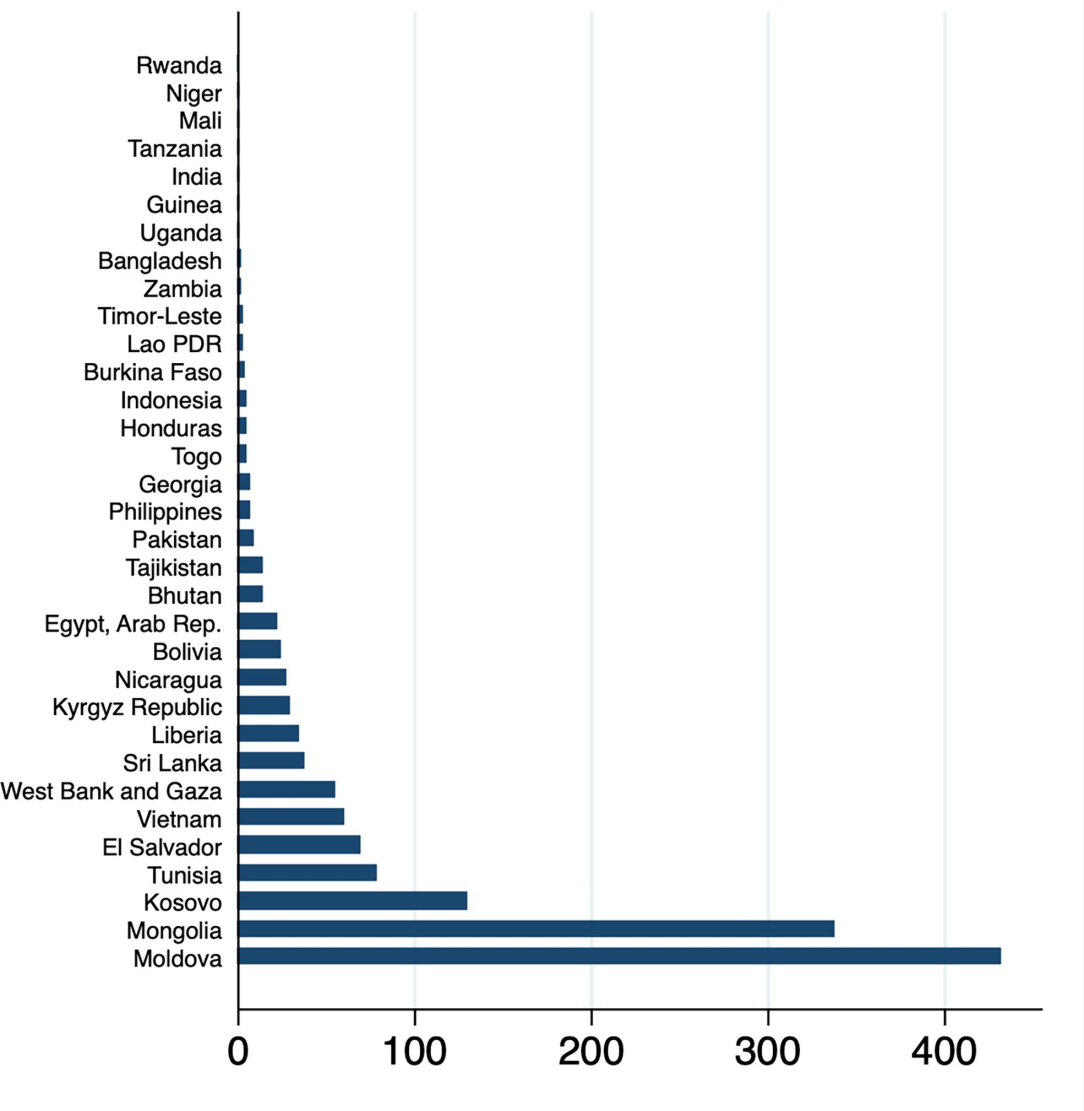
Estimated country-specific costs per poverty case averted using social protection program data. Notes: Source: The World Bank Atlas of Social Protection Indicators of Resilience and Equity (ASPIRE). Sample includes low-income and lower-middle-income countries. Costs are in 1,000 $s.

Next, we use data from the World Bank’s ASPIRE database [10] and estimate the SPL program costs associated with averting poverty. Results (Table 5) show that the derived median cost across all countries (low-income and lower-middle-income countries) is about $8,000 per poverty case averted and that the derived median cost would range from $1,400 per poverty case averted in low-income countries to $25,000 in lower-middle-income countries. Figs 1 and 2 depict the distribution of country-level program costs. We see a very wide distribution in the derived costs per poverty case averted, as demonstrated by the estimates for the 10^th^ and the 90^th^ percentiles in Table 5. Even though the country samples examined are distinct, a comparison of estimates suggests that overall the potential poverty reduction returns through health sector investments (Table 4) could roughly be of the same order of magnitude as the SPL program costs associated with averting poverty (Table 5, Figs 1 and 2).

**Table 4:** Estimations of poverty reduction benchmarks: Cost associated with averting one poverty case, across low-income countries and lower-middle-income countries.

	(1)	(2)	(3)
Country category:	Low-income + Lower-middle income	Low-income	Lower-middle-income
Lower uncertainty bound	$28,480	$250	$38,130
Median estimate	$44,010	$450	$66,460
Mean estimate	$43,630	$460	$67,620
Upper uncertainty bound	$94,160	$1,600	$255,250
# countries	63	20	43

Notes: All estimates are derived using the last year of data available for each country in the sample.

Change in poverty is calculated using the coefficient on lagged per capita health spending from model (1) in which logged poverty is regressed on logged per capita public health spending (lagged one year) while controlling for country fixed effects, country gross domestic product and Gini index (in which poverty headcount is the proportion of a country’s population living on less than $1.90 a day, and public spending per capita for health and gross domestic product per capita are adjusted for purchasing power parity and are in 2017 international dollars). Subsequently, the cost of avoiding one case of poverty is derived.

Source: World Bank World Development Indicators [1].

**Table 5:** Cost ($) per poverty case averted, using social protection program data.

	10th percentile	Median	Mean	90th percentile
Low-income + Lower-middle income	$817	$7,922	$47,130	$85,532
Low-income	$523	$1,449	$6,865	$26,720
Lower-middle-income	$2,223	$24,889	$64,631	$141,674

Source: The World Bank Atlas of Social Protection Indicators of Resilience and Equity (ASPIRE).

All costs are in 2017 international dollars.

## Discussion

We attempt in this paper to conceptualize poverty reduction benchmarks (PRBs) for the health sector, that is the approximate range of costs to avert one poverty case in low- and middle-income country settings. These PRBs could potentially help policymakers assess whether the financial risk protection and poverty reduction benefits of health sector investments yield good value for money (i.e. a large number of poverty cases averted per dollar spent). For example, governments could learn from comparable countries that are able to realize poverty reduction gains at investment levels below the estimated PRBs, and subsequently work towards implementing health interventions that improve not only health outcomes but also perform well in terms of poverty reduction returns.

In our analysis, we first examined the association between poverty headcount and government expenditures on health. Next, we computed the relationship between poverty averted and spending on national social protection programs whose primary intent is to reduce poverty. Comparing the estimates obtained with these two approaches can provide some sense of whether financial protection benefits provided by the health sector are comparable to the poverty reduction benefits achieved through the social protection sector.

We find that a 1.0% increase in government health spending would be associated with a 0.5% reduction in the national poverty headcount ratio across all countries studied. PRBs could provide a sense of how much it costs to avert poverty when funds are funneled through the health sector. The health-specific PRBs we identify are relatively high, which is inevitable given that the primary objective of national health systems is to improve population health; poverty reduction is only a potential secondary intent. The goal for countries would be not just to keep poverty reduction costs below the PRBs, but also to learn from the experiences of countries that lie at the lower end of the distribution of country-specific PRBs (i.e. lower costs per poverty case averted).

Our tentative approach for assessing PRBs is directly inspired by the use of country-level cost-effectiveness thresholds to guide resource allocation decisions in the health sector [11–14]. This work also builds on previous explorations of the relationship between social welfare spending and poverty/deprivation outcomes, many of which have focused on high-income countries [15–18]. A recent analysis that included low- and middle-income countries in its sample used World Bank data to show that among all social protection spending categories, social insurance (primarily public pensions) was best able to improve the living standards of the poorest [19]. The major difference between previous analyses and our work is that we study a spending category (e.g. public expenditures on health) that is much less narrowly targeted than social welfare programs and budgets. We also contribute to the literature on illness-related impoverishment and financial protection though the outcome we examine is all-cause poverty, a category that includes impoverishment due to illness. Finally, our findings complement the evidence base on the impacts of health insurance and universal health coverage on financial outcomes for the poor [20–22].

Our analysis presents a number of important limitations. First, our estimated relationship between poverty and public health spending should not be interpreted as causal. While our model specification was able to account for potential country-specific time invariant sources of bias, many other factors that were not accounted for (e.g. political changes within countries, aid and development assistance) could play a role in the relationship we investigated. We do, however, present results in the supporting information file using a health spending covariate that combines both government and external health expenditures, and these results are broadly consistent with those in the main analysis. Second, while the goal of this paper is to outline an approach that can be used to guide health investments oriented towards poverty alleviation, there is much work to be done to further assess and develop the preliminary method we propose here. For example, it is important to explore different ways of modeling the specification that produces the estimates we use to back out the PRBs, such as the use of dynamic panel modeling techniques [23,24]. Third, our poverty headcount outcome variable (which includes impoverishment due to poor health) presents a number of shortcomings [25]. Yet, alternatives were difficult to implement. For example, actual data on illness-related impoverishment largely rely on country-level imputations [6,7]. Fourth, since our primary aim was to derive PRBs to guide resource allocation decisions toward efficient poverty reduction efforts in low- and middle-income countries, we could have used a variety of approaches to estimate the monetary valuation of averting one case of poverty in such countries. For instance, one could directly ask policymakers how much they would be willing to spend to lift say 100,000 people out of poverty, or implement surveys that incorporate poverty valuation questions. Since individuals invariably vary in how they value poverty alleviation, the results of such surveys are likely to be different across settings and time. While the views and beliefs of multiple policy actors do indeed shape the investments made into a country’s health system at a given point in time, our approach captures estimates of how the health spending-poverty association can play out in reality. Accordingly, our preliminary approach might be viewed as providing a less subjective valuation of poverty reduction. Having said that, we do not call for our approach to supplant other methods, but highlight the preliminary insights it provides when used in conjunction with the rest. Fifth, we note that while our results are likely to approximate the consequences of total government spending in health, we cannot speak to the effects of channeling this spending through different mediums such as OOP payments, pre-payment schemes or insurance mechanisms [6,7]. Sixth, our analysis seeks to inform *current* policymaking by capturing the potential consequences of *past* health investments which were likely shaped by different policy goals and contextual factors. While there invariably are limitations to the extent to which previous experiences can speak to the needs of a different time, our approach uses panel data for a cross-section of countries, and is therefore arguably able to draw broad conclusions about the relationship between health spending and poverty. The results from this kind of exercise are likely to provide a fair sense of how the same relationship might play out in the near future. Finally, it is important to keep in mind that interventions that might not appear to be efficient approaches for poverty reduction could serve other important functions–for example, they might ensure the most vulnerable individuals with a basic level of material wellbeing, or build child human capital. Therefore, policy decisions need to be guided by a plethora of factors, not just health- and poverty-specific efficiency considerations.

In summary, our analysis can be best interpreted as a preliminary attempt to consider the value for money toward poverty reduction in the health sector in the spirit of the cost-effectiveness analysis literature [26,27]. Indeed, similar to cost-effectiveness thresholds, we propose to develop the idea of PRBs, which can help decide when health interventions that can provide financial risk protection may be worthwhile investing in from welfare and social protection standpoints. This would allow policymakers to select the health interventions that can “purchase” financial protection most efficiently. This makes possible the identification of “best-buys” for the health sector in terms of poverty reduction and financial protection [28,29]. Along this line, estimated PRB values could also be compared to health economic evaluation intervention thresholds, in terms of dollar per disability-adjusted life year averted.

## Supporting information

S1 Text (PDF)
