# Supplementary material for: Conceptualizing monetary benchmarks for health investments toward poverty reduction in low- and lower middle-income countries
Source: PLOS Glob Public Health. 2022 Jun 17;2(6):e0000487. doi: 10.1371/journal.pgph.0000487 (PMC10021786; doi:10.1371/journal.pgph.0000487)
Supplement: S1 Text — (PDF) [file pgph.0000487.s001.pdf]

Conceptualizing monetary benchmarks for health investments toward poverty reduction in low- and lower middle-income countries

Averi Chakrabarti, Stéphane Verguet

Department of Global Health and Population, Harvard T.H. Chan School of Public Health, Boston, MA, USA

Supporting Information

Table A: Countries included in main study sample of low- and lower-middle-income countries

	Country	*Income group*	*Region*	*Years included*
1	Angola	Lower middle income	Sub-Saharan Africa	2008, 2018
2	Bangladesh	Lower middle income	South Asia	2005, 2010, 2016
3	Benin	Lower middle income	Sub-Saharan Africa	2003, 2011, 2015
4	Bhutan	Lower middle income	South Asia	2003, 2007, 2012, 2017
5	Bolivia	Lower middle income	Latin America & Caribbean	2002, 2004, 2005, 2006, 2007, 2008, 2009, 2011, 2012, 2013, 2014, 2015, 2016, 2017, 2018, 2019
6	Burkina Faso	Low income	Sub-Saharan Africa	2003, 2009, 2014
7	Burundi	Low income	Sub-Saharan Africa	2006, 2013
8	Cabo Verde	Lower middle income	Sub-Saharan Africa	2007, 2015
9	Cameroon	Lower middle income	Sub-Saharan Africa	2007, 2014
10	Chad	Low income	Sub-Saharan Africa	2003, 2011
11	Comoros	Lower middle income	Sub-Saharan Africa	2004, 2014
12	Congo, Dem. Rep.	Low income	Sub-Saharan Africa	2004, 2012
13	Congo, Rep.	Lower middle income	Sub-Saharan Africa	2005, 2011
14	Cote d'Ivoire	Lower middle income	Sub-Saharan Africa	2002, 2008, 2015

15	Egypt, Arab Rep.	Lower middle income	Middle East & North Africa	2004, 2008, 2010, 2012, 2015, 2017
16	El Salvador	Lower middle income	Latin America & Caribbean	2002, 2003, 2004, 2005, 2006, 2007, 2008, 2009, 2010, 2011, 2012, 2013, 2014, 2015, 2016, 2017, 2018, 2019
17	Eswatini	Lower middle income	Sub-Saharan Africa	2009, 2016
18	Ethiopia	Low income	Sub-Saharan Africa	2004, 2010, 2015
19	Gambia, The	Low income	Sub-Saharan Africa	2003, 2010, 2015
20	Ghana	Lower middle income	Sub-Saharan Africa	2005, 2012, 2016
21	Guinea	Low income	Sub-Saharan Africa	2002, 2007, 2012
22	Guinea-Bissau	Low income	Sub-Saharan Africa	2002, 2010
23	Honduras	Lower middle income	Latin America & Caribbean	2002, 2003, 2004, 2005, 2006, 2007, 2008, 2009, 2010, 2011, 2012, 2013, 2014, 2015, 2016, 2017, 2018, 2019
24	India	Lower middle income	South Asia	2004, 2009, 2011
25	Kenya	Lower middle income	Sub-Saharan Africa	2005, 2015
26	Kyrgyz Republic	Lower middle income	Europe & Central Asia	2002, 2003, 2004, 2005, 2006, 2007, 2008, 2009, 2010, 2011, 2012, 2013, 2014, 2015, 2016, 2017, 2018, 2019
27	Lao PDR	Lower middle income	East Asia & Pacific	2002, 2007, 2012, 2018
28	Lesotho	Lower middle income	Sub-Saharan Africa	2002, 2017
29	Liberia	Low income	Sub-Saharan Africa	2007, 2014, 2016
30	Madagascar	Low income	Sub-Saharan Africa	2005, 2010, 2012
31	Malawi	Low income	Sub-Saharan Africa	2004, 2010, 2016
32	Mali	Low income	Sub-Saharan Africa	2006, 2009
33	Mauritania	Lower middle income	Sub-Saharan Africa	2004, 2008, 2014
34	Micronesia, Fed. Sts.	Lower middle income	East Asia & Pacific	2005, 2013
35	Moldova	Lower middle income	Europe & Central Asia	2002, 2003, 2004, 2005, 2006, 2007, 2008, 2009, 2010, 2011, 2012, 2013, 2016, 2017
36	Mongolia	Lower middle income	East Asia & Pacific	2002, 2007, 2010, 2011, 2012, 2014, 2016, 2018
37	Morocco	Lower middle income	Middle East & North Africa	2006, 2013
38	Mozambique	Low income	Sub-Saharan Africa	2002, 2008, 2014
39	Myanmar	Lower middle income	East Asia & Pacific	2015, 2017

40	Nepal	Lower middle income	South Asia	2003, 2010
41	Nicaragua	Lower middle income	Latin America & Caribbean	2005, 2009, 2014
42	Niger	Low income	Sub-Saharan Africa	2005, 2007, 2011, 2014
43	Nigeria	Lower middle income	Sub-Saharan Africa	2003, 2009, 2018
44	Pakistan	Lower middle income	South Asia	2004, 2005, 2007, 2010, 2011, 2013, 2015, 2018
45	Philippines	Lower middle income	East Asia & Pacific	2003, 2006, 2009, 2012, 2015, 2018
46	Rwanda	Low income	Sub-Saharan Africa	2005, 2010, 2013, 2016
47	Sao Tome and Principe	Lower middle income	Sub-Saharan Africa	2010, 2017
48	Senegal	Lower middle income	Sub-Saharan Africa	2005, 2011
49	Sierra Leone	Low income	Sub-Saharan Africa	2003, 2011, 2018
50	Solomon Islands	Lower middle income	East Asia & Pacific	2005, 2012
51	Sri Lanka	Lower middle income	South Asia	2002, 2006, 2009, 2012, 2016
52	Sudan	Low income	Sub-Saharan Africa	2009, 2014
53	Tajikistan	Low income	Europe & Central Asia	2003, 2004, 2007, 2009, 2015
54	Tanzania	Lower middle income	Sub-Saharan Africa	2007, 2011, 2017
55	Timor-Leste	Lower middle income	East Asia & Pacific	2007, 2014
56	Togo	Low income	Sub-Saharan Africa	2006, 2011, 2015
57	Tunisia	Lower middle income	Middle East & North Africa	2005, 2010, 2015
58	Uganda	Low income	Sub-Saharan Africa	2002, 2005, 2009, 2012, 2016
59	Ukraine	Lower middle income	Europe & Central Asia	2002, 2003, 2004, 2005, 2006, 2007, 2009
60	Uzbekistan	Lower middle income	Europe & Central Asia	2002, 2003
61	Vietnam	Lower middle income	East Asia & Pacific	2002, 2004, 2006, 2008, 2010, 2012, 2014, 2016, 2018
62	Zambia	Lower middle income	Sub-Saharan Africa	2002, 2004, 2006, 2010, 2015
63	Zimbabwe	Lower middle income	Sub-Saharan Africa	2011, 2017, 2019

Table B: Estimating the association between government health spending and poverty alleviation - Government health spending lagged five years

	(1)	(2)	(3)
Dependent variable:	Log poverty headcount ratio		
Country category:	Low-income + Lower-middle-income	Low-income	Lower-middle-income
Log government health spending (lagged five years)	-0.377**	-0.075	-0.558**
	(0.130)	(0.128)	(0.159)
Log GDP per capita	-2.320**	-0.956**	-2.438**
	(0.282)	(0.407)	(0.315)
Gini index	0.046**	0.045**	0.036**
	(0.011)	(0.015)	(0.012)
Country fixed effects	X	X	X
Observations	200	43	157
R-squared	0.97	0.97	0.96
# countries	62	20	42

Standard errors in parentheses. Statistical significance: ***p<0.01, **p<0.05, *p<0.10.

GDP=gross domestic product. Data covers the years 2002-2019. Source: World Development Indicators.

Table C: Using an alternative health spending measure: government + external health spending

Panel A: Estimating the association between health spending (government + external) and poverty alleviation

	(1)	(2)	(3)
Income classification:	Low-income + Lower-middle income	Low-income	Lower-middle-income
Dependent variable:	Log poverty headcount ratio		
Log health spending (government + external)	-0.399**	-0.174	-0.645**
	(0.160)	(0.147)	(0.200)
Log GDP per capita	-2.429**	-0.779**	-2.627**
	(0.287)	(0.331)	(0.332)
Gini index	0.055**	0.037**	0.052**
	(0.010)	(0.012)	(0.011)
Country fixed effects	X	X	X
Observations	273	60	213
R-squared	0.93	0.90	0.92
# countries	63	20	43

Panel B: Estimations of poverty reduction benchmarks: cost associated with averting one poverty case

	(1)	(2)
	Estimated poverty reduction benchmarks	
Country category:	Low-income + Lower-middle income	Lower-middle-income
Lower uncertainty bound	$27,220	$28,070
Median estimate	$50,020	$44,777
Mean estimate	$50,000	$45,290
Upper uncertainty bound	$181,430	$121,300
# countries	63	43

Source: World Development Indicators (WDI).
External health expenditures are from the WDI and include all foreign transfers into a country's health sector. These are funds invested in government schemes or other programs (such as those operated by non-governmental organizations).
Notes for Panel A: Standard errors in parentheses. Statistical significance: ***p<0.01, **p<0.05, *p<0.10. GDP=gross domestic product. Data cover the years 2002-2019.
Notes for Panel B: All estimates are derived using the last year of data available for each country in the sample. Change in poverty is calculated using the coefficient on lagged per capita health spending from model (1) in which logged poverty is regressed on logged per capita public health spending (lagged one year) while controlling for country fixed effects, country gross domestic product and Gini index (in which poverty headcount is the proportion of a country's population living on less than $1.90 a day, and public spending per capita for health and gross domestic product (GDP) per capita are adjusted for purchasing power parity (PPP) and are in 2017 international dollars). Subsequently, the cost of avoiding one case of poverty is derived. We do not present estimations of poverty reduction benchmarks for low-income countries since the main input is statistically insignificant. All costs are in 2017 international dollars.

Table D: Summary statistics - Upper-middle-income countries

	Initial mean	Final mean
Poverty headcount (proportion of country population)	0.09	0.03
Government health expenditures per capita lagged one year	$302	$532
GDP per capita	$10,412	$14,980
Gini index	45	41
N	*37*	

Notes: The poverty line is defined by $1.90 a day in 2011 international prices. Government health expenditures per capita and gross domestic product (GDP) per capita are adjusted for purchasing power parity and are in constant 2017 international dollars.

Table E: Countries included in upper-middle-income country sample

	Country	*Region*	*Years included*
1	Albania	Europe & Central Asia	2002, 2005, 2008, 2012, 2014, 2015, 2016, 2017
2	Argentina	Latin America & Caribbean	2002, 2003, 2004, 2005, 2006, 2007, 2008, 2009, 2010, 2011, 2012, 2013, 2014, 2016, 2017, 2018, 2019
3	Armenia	Europe & Central Asia	2002, 2003, 2004, 2005, 2006, 2007, 2008, 2009, 2010, 2011, 2012, 2013, 2014, 2015, 2016, 2017, 2018, 2019
4	Belarus	Europe & Central Asia	2002, 2003, 2004, 2005, 2006, 2007
5	Bosnia and Herzegovina	Europe & Central Asia	2004, 2007, 2011
6	Botswana	Sub-Saharan Africa	2002, 2009, 2015
7	Brazil	Latin America & Caribbean	2002, 2003, 2004, 2005, 2006, 2007, 2008, 2009, 2011, 2012, 2013, 2014, 2015, 2016, 2017, 2018, 2019
8	Bulgaria	Europe & Central Asia	2006, 2007, 2008, 2009, 2010, 2011, 2012, 2013, 2014, 2015, 2016, 2017, 2018
9	China	East Asia & Pacific	2002, 2005, 2008, 2010, 2011, 2012, 2013, 2014, 2015, 2016
10	Colombia	Latin America & Caribbean	2002, 2003, 2004, 2005, 2008, 2009, 2010, 2011, 2012, 2013, 2014, 2015, 2016, 2017, 2018, 2019
11	Costa Rica	Latin America & Caribbean	2002, 2003, 2004, 2005, 2006, 2007, 2008, 2009, 2010, 2011, 2012, 2013, 2014, 2015, 2016, 2017, 2018, 2019
12	Dominican Republic	Latin America & Caribbean	2002, 2003, 2004, 2005, 2006, 2007, 2008, 2009, 2010, 2011, 2012, 2013, 2014, 2015, 2016, 2017, 2018, 2019
13	Ecuador	Latin America & Caribbean	2003, 2004, 2005, 2006, 2007, 2008, 2009, 2010, 2011, 2012, 2013, 2014, 2015, 2016, 2017, 2018, 2019
14	Fiji	East Asia & Pacific	2002, 2008, 2013
15	Gabon	Sub-Saharan Africa	2005, 2017
16	Georgia	Europe & Central Asia	2002, 2003, 2004, 2005, 2006, 2007, 2008, 2009, 2010, 2011, 2012, 2013, 2014, 2015, 2016, 2017, 2018, 2019
17	Guatemala	Latin America & Caribbean	2006, 2014
18	Indonesia	East Asia & Pacific	2002, 2003, 2004, 2005, 2006, 2007, 2008, 2009, 2010, 2011, 2012, 2013, 2014, 2015, 2016, 2017, 2018, 2019
19	Iran, Islamic Rep.	Middle East & North Africa	2005, 2006, 2009, 2013, 2014, 2015, 2016, 2017, 2018
20	Iraq	Middle East & North Africa	2006, 2012
21	Jamaica	Latin America & Caribbean	2002, 2004

22	Jordan	Middle East & North Africa	2002, 2006, 2008, 2010
23	Kazakhstan	Europe & Central Asia	2002, 2003, 2004, 2005, 2006, 2007, 2009, 2010
24	Malaysia	East Asia & Pacific	2003, 2006, 2008, 2011
25	Maldives	South Asia	2002, 2009
26	Mexico	Latin America & Caribbean	2002, 2004, 2005, 2006, 2008, 2010, 2012, 2014, 2016, 2018
27	Namibia	Sub-Saharan Africa	2003, 2009, 2015
28	North Macedonia	Europe & Central Asia	2009, 2010, 2011, 2012, 2013, 2014, 2015, 2016, 2017, 2018
29	Paraguay	Latin America & Caribbean	2002, 2003, 2004, 2005, 2006, 2007, 2008, 2009, 2010, 2011, 2012, 2013, 2014, 2015, 2016, 2017, 2018, 2019
30	Peru	Latin America & Caribbean	2002, 2003, 2004, 2005, 2006, 2007, 2008, 2009, 2010, 2011, 2012, 2013, 2014, 2015, 2016, 2017, 2018, 2019
31	Russian Federation	Europe & Central Asia	2002, 2003, 2004, 2005, 2006, 2007, 2008, 2009, 2010
32	Samoa	East Asia & Pacific	2002, 2008, 2013
33	Serbia	Europe & Central Asia	2012, 2013, 2014, 2015, 2016, 2017
34	South Africa	Sub-Saharan Africa	2005, 2008, 2010, 2014
35	Thailand	East Asia & Pacific	2002, 2004, 2006, 2007, 2008, 2009, 2010, 2012, 2019
36	Tonga	East Asia & Pacific	2009, 2015
37	Turkey	Europe & Central Asia	2002, 2003, 2004, 2005, 2006, 2007, 2008, 2009, 2010, 2011, 2012, 2013, 2014, 2015, 2016, 2017, 2019

Table F: Results for the sub-sample of upper-middle-income countries

Estimating the association between government health spending and poverty alleviation			
Dependent variable:	Log poverty headcount ratio		
	(1)	(2)	(3)
Main covariate lagged by:	One year	Five years	One year
Type of health spending covariate	Government spending	Government spending	Government + external spending
Log health spending	-0.104	-0.285**	-0.250*
	(0.142)	(0.128)	(0.142)
Log GDP per capita	-2.610**	-2.645**	-2.406**
	(0.275)	(0.323)	(0.257)
Gini index	0.084**	0.066**	0.081**
	(0.011)	(0.013)	(0.011)
Country fixed effects	X	X	X
Observations	347	265	347
R-squared	0.90	0.94	0.90
# countries	37	36	37

Notes: Standard errors in parentheses. Statistical significance: ***p<0.01, **p<0.05, *p<0.10. GDP=gross domestic product. Data cover the years 2002-2019. Source: World Development Indicators.
We do not present estimations of poverty reduction benchmarks using public health expenditure data (as in Table 4 and Table S3) for upper-middle-income countries since the main inputs are statistically insignificant or only marginally significant.
All costs are in 2017 international dollars.
